# Preventing intrusive memories after trauma via a brief intervention involving Tetris computer game play in the emergency department: a proof-of-concept randomized controlled trial

**DOI:** 10.1038/mp.2017.23

**Published:** 2017-03-28

**Authors:** L Iyadurai, S E Blackwell, R Meiser-Stedman, P C Watson, M B Bonsall, J R Geddes, A C Nobre, E A Holmes

**Affiliations:** 1 Department of Psychiatry, University of Oxford, Oxford, UK; 2 Medical Research Council Cognition and Brain Sciences Unit, Cambridge, UK; 3 Department of Clinical Psychology and Psychotherapy, Ruhr-Universität Bochum, Bochum, Germany; 4 Department of Clinical Psychology, University of East Anglia, Norwich, UK; 5 Department of Zoology, University of Oxford, Oxford, UK; 6 Oxford Health NHS Foundation Trust, Oxford, UK; 7 Division of Psychology, Department of Clinical Neuroscience, Karolinska Institutet, Stockholm, Sweden

## Abstract

After psychological trauma, recurrent intrusive visual memories may be distressing and disruptive. Preventive interventions post trauma are lacking. Here we test a behavioural intervention after real-life trauma derived from cognitive neuroscience. We hypothesized that intrusive memories would be significantly reduced in number by an intervention involving a computer game with high visuospatial demands (Tetris), via disrupting consolidation of sensory elements of trauma memory. The Tetris-based intervention (trauma memory reminder cue plus c. 20 min game play) vs attention-placebo control (written activity log for same duration) were both delivered in an emergency department within 6 h of a motor vehicle accident. The randomized controlled trial compared the impact on the number of intrusive trauma memories in the subsequent week (primary outcome). Results vindicated the efficacy of the Tetris-based intervention compared with the control condition: there were fewer intrusive memories overall, and time-series analyses showed that intrusion incidence declined more quickly. There were convergent findings on a measure of clinical post-trauma intrusion symptoms at 1 week, but not on other symptom clusters or at 1 month. Results of this proof-of-concept study suggest that a larger trial, powered to detect differences at 1 month, is warranted. Participants found the intervention easy, helpful and minimally distressing. By translating emerging neuroscientific insights and experimental research into the real world, we offer a promising new low-intensity psychiatric intervention that could prevent debilitating intrusive memories following trauma.

## Introduction

After psychological trauma, sensory memories can recurrently spring to mind unbidden,^1^ bringing back sights and sounds of the events, evoking strong emotion, hijacking attention and profoundly disrupting current activities. Intrusive memories comprise a core clinical feature^2^ of acute stress disorder and post-traumatic stress disorder (PTSD).^1^ In the first days post trauma, intrusive memories (among other symptoms) have been associated with a diagnosis of PTSD at 1 year,^3^ and early intrusion symptoms with a non-remitting PTSD trajectory over 15 months.^4^ Intrusive memories also occur across a range of other mental disorders from depression^5^ to complicated grief,^6^ comprising an important transdiagnostic target^7^ for preventive psychiatric interventions. However, currently preventive interventions after trauma targeting the full syndrome of PTSD are either ineffective^8, 9^ or unappealing/inaccessible^10, 11^ to most people. New approaches are needed—we suggest targeting preventive efforts on a focal symptom—here, intrusive memories of the trauma.

We have called for the development of mechanistically driven behavioural interventions,^12^ preferably low-intensity and deliverable by non-specialists to allow for scalability.^13^ A potential treatment opportunity as a cognitive 'therapeutic vaccine' (ref 14, p 1315) delivered post trauma^15^ to limit the reoccurrence of intrusive memories is offered by a combination of insights from neuroscience. Memory consolidation theory suggests a time window of several hours post trauma during which trauma memory is malleable and vulnerable to disruption.^16, 17^ Animal research examining molecular and cellular processes in memory consolidation demonstrates that it is possible to interfere with fear memory stabilization soon after learning, for example, using the protein synthesis inhibitor anisomycin.^18, 19, 20^ In humans, however, protein synthesis inhibitors are toxic: studies using the β-blocker propranolol as a consolidation blockade have not yet shown success,^21, 22^ and electroconvulsive therapy, while effective in disrupting memory,^23^ cannot be used readily in the aftermath of trauma. Techniques are needed to target the *intrusive* nature of memories specifically as this is associated with clinical impairment. Cognitive science predicts that cognitive tasks with high visuospatial demands will selectively disrupt sensory (predominantly visual) aspects of memory (i.e. those that underpin intrusions) via competition for limited cognitive resources^24, 25, 26^ when that memory is labile. Combined, these insights suggest that engaging in cognitive tasks with high visuospatial demands during the time window of trauma memory consolidation may reduce the occurrence of subsequent intrusive visual memories of trauma.

Using an experimental analogue of a traumatic event (film footage) under controlled laboratory settings, we have tested the relative efficacy of various cognitive tasks in reducing intrusive memories. In line with the hypotheses, the results show that visuospatial tasks (e.g. complex pattern tapping) during or soon after the event consistently lead to a reduction in the number of subsequent intrusive memories,^15, 27, 28, 29, 30^ whereas some verbal tasks (e.g. backwards counting; the verbal computer game Pub Quiz) do not and can even increase intrusions indicating possible harmful effects^15, 27, 31, 32^ (though see Van den Hout and Engelhard^33^). We suggest that visuospatial cognitive tasks act not merely via distraction, but by modality-specific interference with sensory (visual) aspects of intrusive memory. Critical to future translation to real trauma, we have shown that a popular and widely available visually absorbing computer game (playing 'Tetris')^34^ is also effective as a component of a behavioural intervention compared with control conditions (a verbal 'Pub Quiz' game or no task).^15, 29, 35^

In a parallel randomized controlled trial, we tested the hypothesis that a behavioural intervention (playing the visually absorbing computer game Tetris after a reminder cue), compared with an attention-placebo (ref 36, p 191) control condition (a written activity log), would reduce the number of intrusive memories of trauma over the subsequent week. The control condition was selected for nonspecific factors including time, contact with the researcher, location of treatment procedure and engagement in a structured task. Unlike the intervention condition, the control condition contained no reminder cue for the traumatic event. Study information and materials referred to 'simple activities' in both conditions for credibility (Supplementary Information). The primary outcome was the number of intrusive memories 1 week post accident (as logged in a daily diary); secondary outcomes were post-trauma symptomatology, anxiety and depression (1 week/1 month). Participant feedback was assessed at 1 month.

## Materials and methods

### Participants and procedure

**Figure 1:**
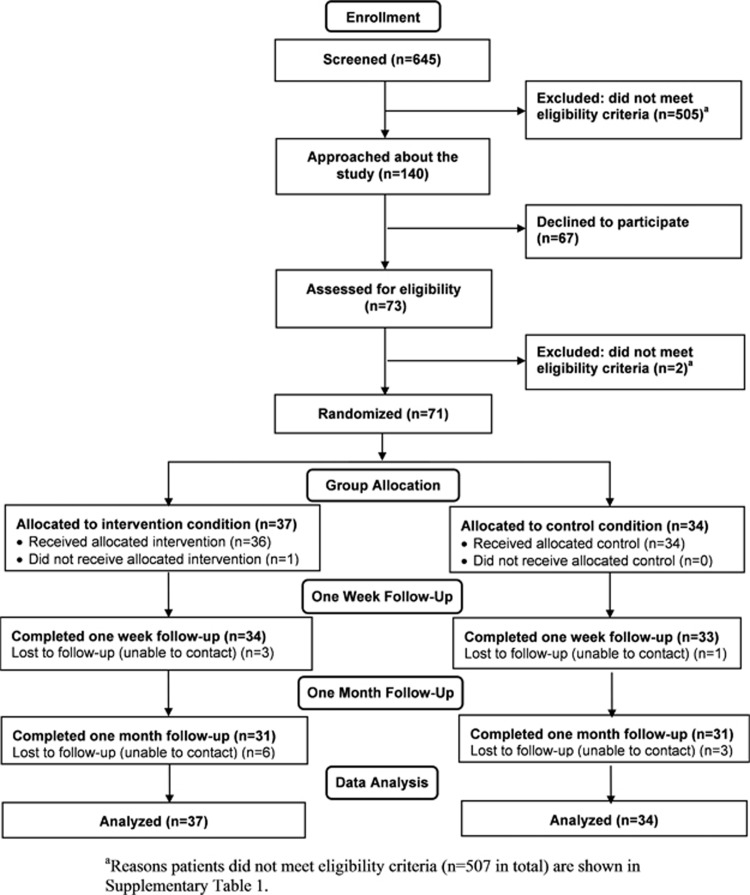
CONSORT participant flow diagram for the trial.

Participants were 71 patients presenting to the emergency department of the John Radcliffe Hospital (Oxford, UK) (Figure 1): 34 were men and 37 were women, and the mean age was 39.66 years (s.d. 16.32). Inclusion criteria were: age ⩾18 years; experienced/witnessed a motor vehicle accident (as a driver, passenger, motorcyclist or pedestrian); met Diagnostic and Statistical Manual of Mental Disorders 4th Edition (DSM-IV) PTSD criterion A1 for a traumatic event ('experienced, witnessed or was confronted with an event or events that involved actual or threatened death or serious injury'); seen in emergency department within 6 h of leaving scene of the accident; reported memory of the accident; fluent in written and spoken English; alert and orientated, Glasgow Coma Scale^37^ score=15; and sufficient physical mobility to play a computer game on the intervention platform (Nintendo DS) at the point of taking informed consent. Exclusion criteria were loss of consciousness for >5 min, reported history of severe mental illness, current intoxication, substance abuse or neurological condition, or currently suicidal. The study was approved by the local National Research Ethics Service Research Ethics Committee (Oxford C: 12/SC/0485).

Potential participants were identified by emergency department staff. Eligibility was assessed by a clinical psychologist (LI) using information from medical records and face-to-face interview. After receiving a description of the study, all participants provided written informed consent before completing baseline measures. Participants were then randomly allocated in a 1:1 ratio to two parallel treatment conditions using a web-based randomization system, provided by the Oxford Cognitive Health and Neurosciences Clinical Trials Unit and verified by independent statisticians. Randomization was used to balance groups on known and unknown baseline predictors of outcome,^38^ and carried out using minimization^39^ based on gender, age and perceived life threat to self, with an additional random component to ensure allocations remained unpredictable. The randomization system was accessed by the researcher (LI) from a separate office after baseline measures had been completed. Participants were not informed as to condition allocation. The researcher delivering the procedures (LI) was not blind to participant allocation, as the need to provide verbal instructions precluded such blinding.

Outcome assessment was scheduled for 1 week and 1 month after the accident. After 1-month follow-up, participants were contacted by telephone and debriefed. They were offered a £30 GBP ($45 USD) store voucher to compensate them for their time. Recruitment occurred between March 2014 and January 2015. The last follow-up assessment was completed in February 2015. The trial ended once the planned sample size had been achieved.

### Assessments

#### Traumatic event and emergency department treatment characteristics

Details of participants’ traumatic event, treatment in the emergency department and previous emergency department attendances were collected from medical records. Severity of physical injury, indicated by the Injury Severity Score (range 0–75), was rated using the Abbreviated Injury Scale.^40^ Agreement for injury codings between raters (LI and a research nurse) was 100%. Participants rated perceived life threat during the accident: 'to what extent did you feel your life was in danger?' and 'to what extent did you feel that someone else’s life was in danger?' from 0 (not at all) to 10 (extremely).^41^ Dissociative symptoms were assessed with the Peritraumatic Dissociative Experiences Questionnaire-Self Report.^42^ Emotional responses were assessed with the Peritraumatic Distress Inventory.^43^ Participants reported experience of prior psychological trauma, current and past mental illness, and family history of mental illness.

#### Primary outcome

The total number of intrusive memories in the week after the traumatic event was assessed using a daily pen-and-paper diary (adapted from refs 15, 29, 35). Participants recorded the occurrence of intrusive memories in everyday life by ticking a box for the day and time period (morning/afternoon/evening) when the intrusive memory occurred, or marked 'zero' if they experienced none. Intrusive memories were described as: 'image-based memories of the accident that pop into your mind without warning. They often take the form of visual pictures in your mind’s eye, for example, like a snapshot image or a film clip. They can also include other senses, for example, sounds and smells'. Participants were not to record memories recalled deliberately or general verbal thoughts. For examples of intrusive memories see Supplementary Table 2. The diary started on the day of the accident ('Day 1') and was completed for seven days. Daily reminders to complete the diary were sent via SMS. Upon completion, participants rated 'how accurately do you think you completed the diary?' from 0 (not at all) to 10 (extremely). Participants returned the diary by post. The *number* of intrusive memories was selected for several reasons: it allows comparison with preclinical work; was readily understood by traumatized patients without the need for explanation from an expert; and is directly relevant to the clinical goal of reducing the number of times the memory intruded (not just reducing its intensity).

#### Secondary outcomes

Post-trauma distress was assessed using the Impact of Event Scale—Revised,^40, 45^ which has subscales for intrusion, avoidance and hyperarousal symptoms. PTSD symptom severity was assessed with the Post-traumatic Diagnostic Scale (PDS; Foa^46^). Anxiety and depression symptoms were assessed with the Hospital Anxiety and Depression Scale.^47^ To minimize assessor bias and participant burden, measures were completed remotely (online using the secure web-based software 'Qualtrics'^48^ or by post).

#### Participant feedback

A 13-item feedback questionnaire assessed participants’ experience of the study. Items included ratings of how easy, helpful and distressing/burdensome participants found playing Tetris on a scale from 1 (not at all) to 9 (extremely), expectancy ratings for their condition on number of intrusive memories on a 21-point scale from −10 (extreme decrease), 0 (no effect) to +10 (extreme increase)^35^ and open questions.

### Treatment conditions

Participants in both treatment conditions received usual care in the emergency department (e.g. assessments and medical treatment). The condition-specific procedures below were delivered around usual care by a clinical psychologist (LI). Both condition-specific procedures were standardized and structured.

#### Intervention

The intervention procedure involved two key components: a reminder cue for the traumatic event followed by playing the computer game Tetris.^29^ For the memory reminder cue, participants were asked to think back to the accident and briefly tell the researcher the worst moments that came to mind.^49^ Following instructions and practice, participants played Tetris on a Nintendo DS XL. Participants were required to undertake minimum Tetris game play for at least one uninterrupted period of 10 min and for ~20 min in total.

#### Control

In the control procedure participants filled in a simple activity log to note down each activity they had already engaged in during their time in the emergency department. They wrote brief entries in a list in a column (e.g., reading, talking, receiving treatment, crossword, texting) and recorded each activity’s duration (in minutes) in a second column. Following instructions of how to complete the log, participants completed it using pen-and-paper on a clipboard for ~20 min (a similar duration to the intervention condition).

Time spent playing Tetris and filling the activity log was equivalent; and total time spent in the emergency department, during which other activities were unrestricted, was equivalent in the two conditions (Supplementary Information).

### Data analysis

#### Power analysis

Using a conservative estimated effect size of Cohen’s *d*=0.7, based on a previous laboratory study,^29^ a total sample size of *n*=66 was required to provide 80% power at *α*=0.05, two-tailed. Recruitment of at least 70 participants was planned to allow for attrition.

#### Main efficacy analyses

Analyses were intention-to-treat, including all randomized participants. Missing values were estimated using multiple imputation. Five data sets were generated for each missing value at 1 week follow-up, and 10 data sets for each missing value at 1-month follow-up, in line with the recommendation that the number of data sets approximates the percentage of missing data.^50^ Treatment group, age, gender and perceived life threat to self were included as auxiliary variables.^51^ Estimates were pooled in line with guidelines for multiple imputation.^52^ As all between-group comparisons in continuous outcomes at 1 week and 1 month were planned *a priori*, differences were tested using two-tailed *t*-tests at *α*=0.05 (ref 53, p 372). Cohen’s *d* effect sizes were calculated as *t*[Sqrt(1/*n*1+1/*n*2)], and 95% confidence intervals for the effect size were calculated using ESCI software.^54^ Percentage of participants with a symptom profile on the PDS consistent with a DSM-IV diagnosis of PTSD was compared between conditions using logistic regression (2 × 2 *χ*^2^ test) at *α*=0.05, two-tailed. Efficacy analyses were conducted in SPSS version 22^55^ by the first author and verified by a statistician (PCW) blind to condition. Before analysis, 100% of raw data for the primary efficacy analysis, and a randomly selected 10% of raw data for the secondary efficacy analyses, were checked for accuracy by a researcher not involved in data collection and blind to condition. Analyses were also conducted on a 'per-protocol' population (Supplementary Table 3).

#### Exploratory analyses

To investigate the time course of intrusive memories reported in the daily diary over the first seven days after the accident, frequency scattergraphs showing the distribution of the number of intrusive memories on each day per condition were plotted. A nonlinear time-series analysis was used to produce a nonparametric line of best fit, summarizing the distribution of the number of intrusive memories on each day, smoothed from day to day over the 7-day period, by accounting for the number of intrusive memories at nearby time points (autocorrelation). This was achieved by fitting counts of the number of intrusive memories for each participant (*Y*) through time (*t*) with a generalized additive model:^56^


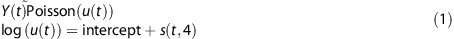


where *u* is a random variable of time and *s*(*t*, 4) is the smoother with four effective degrees of freedom (as in James *et al.*^35^). Expected Poisson distributions at day 2 and day 7 were generated. Time-series analyses were undertaken in R using data provided by participants who returned the diary (*n*=67).

#### Code availability

The computer code used to generate the results can be accessed via Open Science Framework at https://osf.io/e4hc7.

## Results

### Participants

Figure 1 shows the participant flow diagram. Table 1 provides sample, traumatic event and emergency department treatment characteristics for the intervention and control conditions, with no significant differences between conditions.

### Treatment adherence, attrition and adverse effects

All participants allocated to the intervention condition completed the memory reminder cue, and only one participant did not play Tetris for the minimum required duration of 10 min uninterrupted (they were moved by staff to a different bay). All participants allocated to the control condition completed the activity log. Self-report accuracy ratings for completion of the daily diary were high and did not differ between conditions. Attrition (whole sample) was 6% at 1 week and 13% at 1 month. Further details regarding diary completion accuracy ratings and attrition are provided in Supplementary Information. No adverse effects (i.e., negative reactions to the treatment procedures, such as significantly increased distress or suicidality) were recorded in either condition.

### Main efficacy analyses

Continuous outcomes were transformed to correct for skewness using the natural logarithmic function with the exception of the 1-week Impact of Event Scale—Revised intrusion subscale score (which approximated a normal distribution). Variances were similar between conditions on all outcomes. Table 2 shows intention-to-treat analyses for primary and secondary outcomes. Per-protocol analyses showed the same pattern of results (Supplementary Table 3).

#### Primary outcome

**Figure 2:**
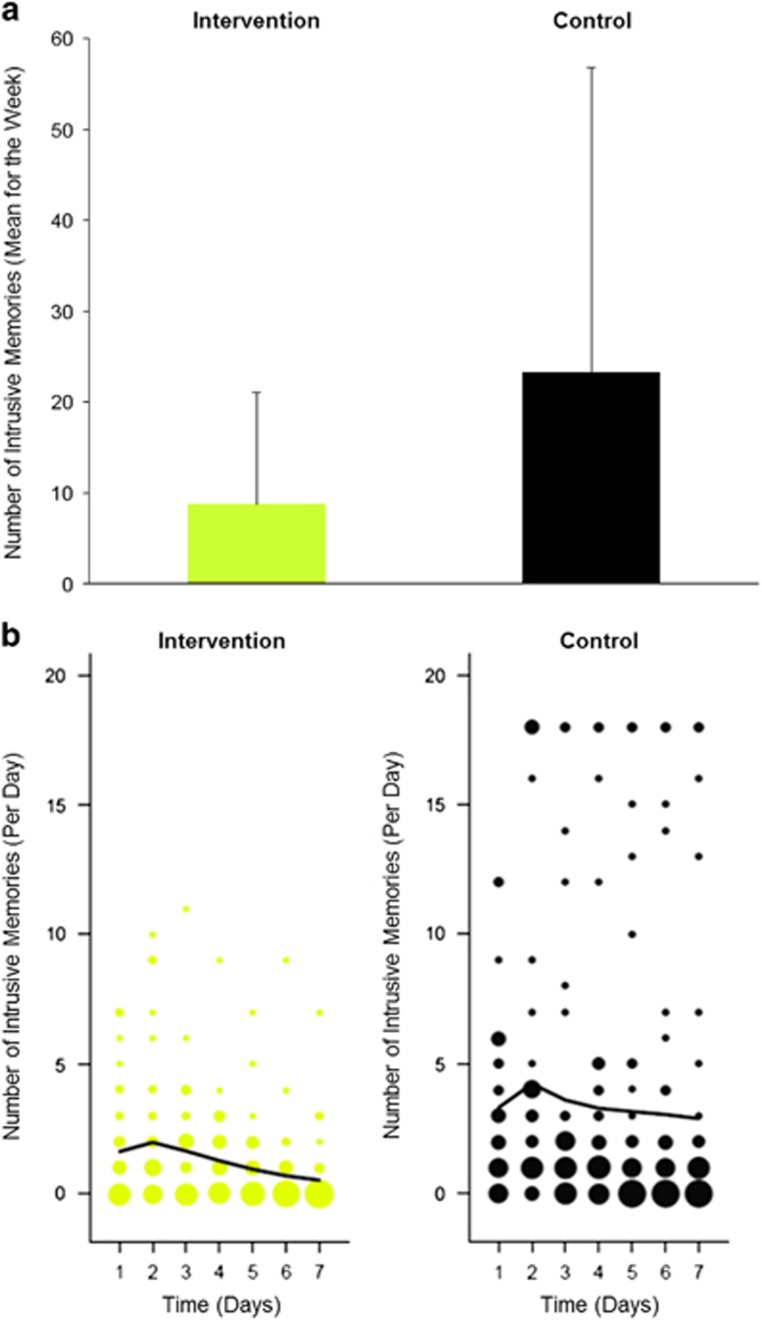
Number of intrusive memories of the traumatic event in the intervention and control conditions. (**a**) Mean number of intrusive memories recorded in a daily diary during the week following a traumatic motor vehicle accident (intention-to-treat analysis). Intervention condition=cognitive task (trauma memory reminder cue plus Tetris computer game play); Control condition=written activity log. There was a significant difference between the intervention condition (*n*=37, *M*=8.73, s.d.=11.55, range 0–55) and the control condition (*n*=34, *M*=23.26, s.d.=32.99, range 0–120): *t*(69)=2.80, *P*=0.005, *d*=0.67, 95% CI: 0.18, 1.14. Error bars show standard deviations. (**b**) Frequency scattergraphs (exploratory analysis) showing the time course of the number of intrusive memories recorded in a diary from day 1 (day of trauma) to day 7 for participants who returned the diary in the intervention condition (*n*=34) and control condition (*n*=33). The size of the circles represents the number of participants who reported the indicated number of intrusive memories on that particular day, scaled separately for each condition. The solid lines are the fit of the generalized additive model (see Equation (1)) to summarize the number of intrusive memories through the 7-day period.

Participants in the intervention condition recorded significantly fewer intrusive memories in the week after the accident than participants in the control condition, with a medium effect size^57^ (*M*=8.73 vs *M*=23.26, *t*(69)=2.80, *P*=0.005, *d*=0.67, 95% CI: 0.18,1.14) (Figure 2a).

#### Secondary outcomes

At 1 week follow-up, participants in the intervention condition reported less distress from intrusion symptoms (Impact of Event Scale—Revised intrusion subscale) than participants in the control condition, with a medium effect size (Table 2). Effect sizes for all other measures at one week, and all measures at one month, were small to negligible (Table 2).

### Exploratory analyses

**Figure 3:**
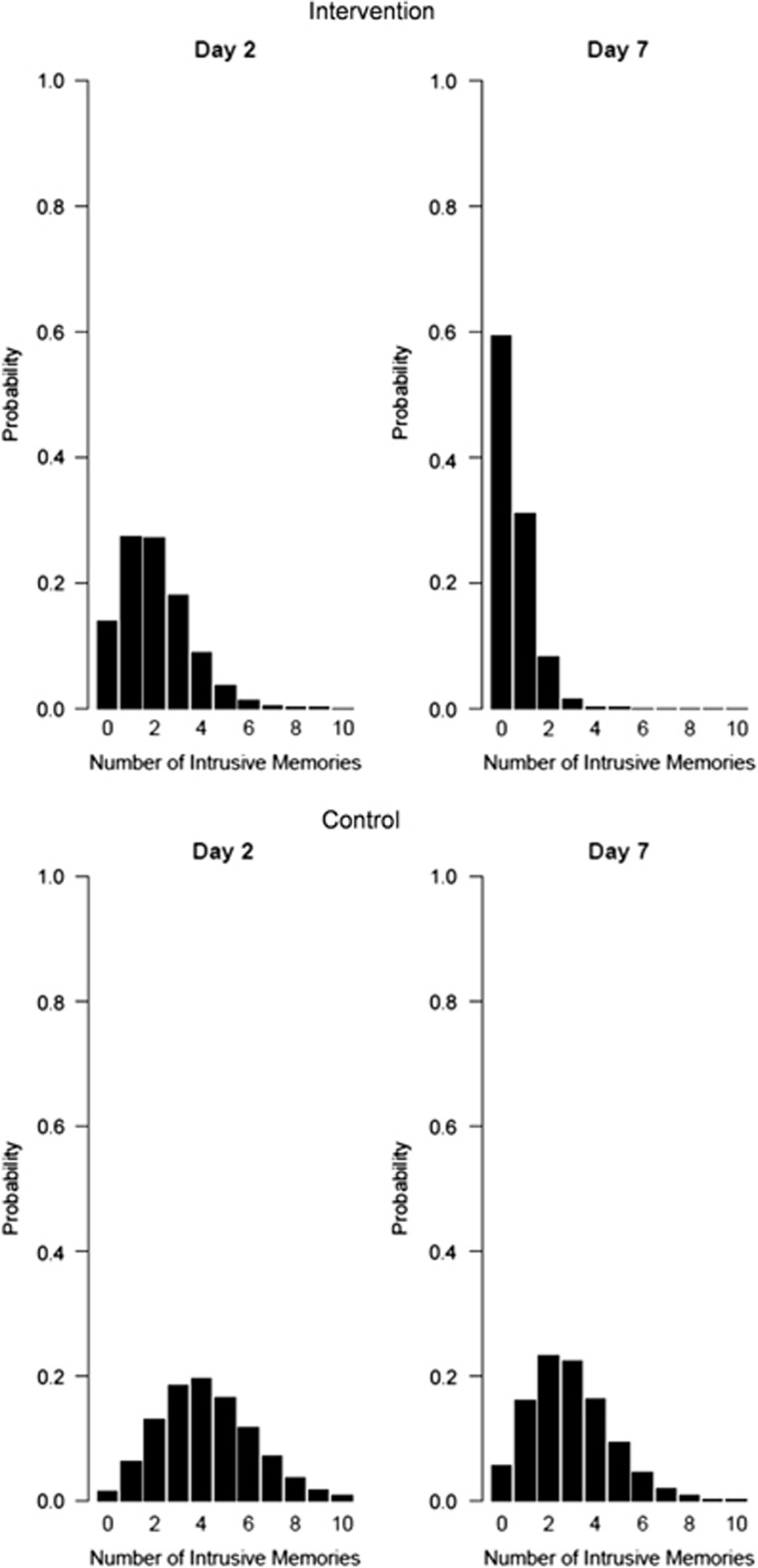
Poisson distributions of the expected probability of the number of intrusive memories at day 2 and day 7 after a traumatic motor vehicle accident in the intervention condition (*n*=34; top row) and the control condition (*n*=33; bottom row) (for participants who returned the diary), showing an advantage of the intervention condition.

From nonlinear time-series analysis, the generalized additive model summarizing the distribution of the number of intrusive memories on each of the first seven days after the accident indicated fewer intrusive memories across the seven days, as well as a more marked decline over the seven days, in the intervention compared with the control condition (Figure 2b). Poisson distributions revealed that the expected probability of having *zero* intrusive memories showed a greater increase over time for participants in the intervention condition (from 13.8% on day 2 to 59.3% on day 7) compared with participants in the control condition (from 1.5% on day 2 to 5.6% at day 7) (Figure 3).

### Participant feedback

Feedback ratings indicated that participants in the intervention condition found playing Tetris very easy (median=7), very helpful (median=7) and minimally distressing/burdensome (median=1).

Examples of participants’ experience of the intervention follow:

One woman in her 20s described having repeated intrusive memories of her motor vehicle accident trauma: 'the picture of falling on the street with my head kept popping up in my head' and 'seeing blood dripping'. She engaged well with playing Tetris in the emergency department and found it fun. 'I think it helped a lot to distract my mind after [the] accident by playing Tetris'.

A woman in her 60s who had never played Tetris before, nor used a Nintendo DS, described playing the game as 'good, really fun'. She asked to continue playing for a little longer, even when the 20-min intervention period was over. At the end of the study, she commented: 'It certainly took my mind off of it at a time when I probably would have sat brooding and feeling very sorry for myself… when you're running the whole thing through your mind and you're on your own at a vulnerable time after the ambulance crew have left you'.

One man in his late 30s described intrusive images of seeing the tree just before the moment of collision, followed by the white flash of the airbag. He wrote at the end of the study: 'I think that playing Tetris helped focus my mind and bring some 'normality' back to my head. I didn't dwell on the accident too much while I was in hospital. Playing Tetris seemed a bit strange at the time, but looking back it has been a help. Thank you'.

## Discussion

An experimental science-driven intervention to reduce intrusive memories of real-world trauma, in patients presenting to a hospital emergency department after a traumatic motor vehicle accident involving directly experienced actual or threatened death or serious injury, was effective compared with an attention-placebo control. This hypothesis-driven work is at the interface between preclinical and clinical research in psychiatry, psychology and post-traumatic reactions. Its principles are derived from molecular, cellular and neural research on memory consolidation^16, 18, 19, 20^ alongside the neuroscience of visual memory,^58^ deploying a behavioural technique to modify emotional intrusive memory. Results provide a critical and compelling translation of previous laboratory findings with experimental trauma^15, 29, 35^ to the ‘real world’. The behavioural intervention with traumatized individuals comprised two steps: (i) a trauma memory reminder cue, followed by (ii) engaging for at least 10 min in a computer game with high visuospatial demands (Tetris)—hypothesized to compete with consolidation of visual memories of trauma. The intervention reduced the number of intrusive memories by 62% in the subsequent week compared with control.

This result with patients compares favourably to previous laboratory-based trauma simulation studies, which found, for example, a relative reduction in intrusive memory count by 58% over the same time period.^29^ Time-series analyses revealed an accelerated recovery from intrusive memories over the first week in the intervention condition, mirroring laboratory findings.^35^ There were convergent findings on a measure of clinical post-trauma intrusion symptoms (Impact of Event Scale—Revised intrusion subscale) at 1 week, but not on other symptom clusters or at 1 month. The current study was designed to detect an effect on the primary outcome measure at 1 week; results suggest that a larger trial, powered to detect differences at 1 month, is warranted.

This brief 'therapist-free' technological intervention was found to be feasible and acceptable—with 48% of patients approached agreeing to participate (compared with 10% in a psychotherapy trial^11^ and 8% in a pharmacological trial,^21^ both also in the emergency department), and with intervention completion at 97%. No adverse effects were observed or reported. Comparison with the wider population of motor vehicle accident survivors seen in the emergency department during the period of data collection suggested our sample had a greater severity of physical trauma (Supplementary Table 4). Patients were reached within a short time post trauma (~3 h after their motor vehicle accident), that is, within a memory consolidation time window as planned.

Our experimental medicine approach targets one core clinical feature—intrusive memories of trauma—rather than a whole psychiatric syndrome (ref 59, p 22). We found effects specific to this subset of symptoms (cf. Soeter and Kindt^60^), that is, those related to the hypothesized mechanisms of the intervention. After a comprehensive therapy for full PTSD such as trauma-focussed cognitive therapy, symptom clusters change together;^61^ that our data suggests the possibility that specific symptom subsets may be targeted raises intriguing questions about the disorder's heterogeneity and inner structure.^62^ The distinction between *intrusive* vs voluntarily recalled memories is important for treatment development. The critical difference between clinical and non-clinical memories of a traumatic event is that the former spring to mind unbidden (i.e. are *intrusive*), and not that a memory of the traumatic event exists *per se*. Indeed, we have argued that 'erasure' of the whole trauma memory could be undesirable,^63^ as patients may need to deliberately remember events, for example, for legal testimony.

Preventive interventions post trauma are currently lacking. Motor vehicle accidents are common traumatic events,^64^ and after-effects in terms of mental disorder well established.^65, 66^ In the emergency department, patients typically wait up to 4 h or more in the United Kingdom,^67^ providing an opportunity to reach patients within a few hours of a traumatic event, that is, within the putative timeframe for memory consolidation. The feasibility of implementing the intervention^68^ is promising due to its brevity, low cost, simplicity for training and delivery, and flexibility of administration (Supplementary Information).

A brief, science-driven intervention offers a low-intensity means that could substantially improve the mental health of those who have experienced psychological trauma—and for we believe the first time offers a cognitive 'therapeutic vaccine' (ref 14, p 1315) that could be administered soon *after* a traumatic event (cf. rabies vaccine after a dog bite) to *prevent* intrusive memories of trauma in the subsequent week. It will be important to build on the findings of this study to understand which intervention aspects are critical. For example, our intervention comprised both a trauma memory reminder and Tetris game play, in line with our aim of translating to a clinical setting our earlier laboratory findings during a putative time window for reconsolidation^35^ and consolidation.^15, 29^ The choice of including a reminder cue during memory consolidation may be surprising, but recent experimental work suggests that even then a cue may be critical.^69^ We reasoned that reminder cues serve additional functions beyond reactivation and rendering memory labile—such as orienting the interference procedure to more targeted elements within the memory trace. This idea is relevant for complex memories,^70, 71^ here visual elements of complex real-world trauma scenes. In contrast, in animal studies in which pharmacological agents block the protein synthesis necessary for memory consolidation,^19^ such blockade is likely to affect multiple elements of a freshly encoded experience, rather than one specific aspect. Further work is needed to unpick this mechanism. We hypothesize that not only Tetris but any task with high visuospatial demands is likely to be useful within the procedure (e.g. games such as Candy Crush, drawing) unlike predominantly verbally distracting tasks (e.g. reading, crosswords). However, either element of the intervention in isolation (memory reminder/visuospatial task) is unlikely to be effective.^35^

This is an early-phase or explanatory trial to establish the efficacy of the intervention (ref 72, p 23), and the activity log was selected to control for as many nonspecific confounding factors as possible, while minimizing the potential for harmful effects.^15, 31^ There is no comparator preventive treatment in the immediate aftermath of trauma^8, 9, 73^ to use as a control. Future mechanism-focussed research should seek improved controls: this requires innovation, as in comparison with drug trials, for psychological interventions it is challenging to create an 'inactive' control that resembles the active treatment.^74^ While it is not possible to blind participants in psychological trials, in the current study expectations do not appear to be associated with performance on the primary outcome measure (Supplementary Information).

Preventive mental health interventions are needed post trauma. Here we find a positive effect of the Tetris-based intervention delivered soon after trauma in the emergency department on the primary outcome measure—intrusive memories of trauma over 1 week. Future studies are needed, designed to test whether effects extend to 1 month or longer. Only one 'dose' of the 20 min intervention was given, and the opportunity to give multiple doses and/or longer durations should be explored. The clinical utility to patients of a brief intervention that reduces their symptoms even if limited to the first week post trauma should also be explored. Critically, as yet successful translations of contemporary neuroscience into mental health treatments have been lacking. This study illuminates how combining clinical (trauma), neuroscientific (memory consolidation) and cognitive (cognitive task competition) theory can lead to a novel and effective clinical technique—opening the way for other mechanistically driven behavioural treatment innovations.

## Figures and Tables

**Table 1:** Sample, traumatic event and emergency department treatment characteristics of study participants by treatment condition

*Sample characteristics*	*Intervention (*n*=37)*		*Control (*n*=34)*	
	*Mean*	*S.d.*	*Mean*	*S.d.*
Age (years)	38.9	16.1	40.5	16.8
Years in education	15.9	3.3	15.2	3.2
				
	n	%	n	%
*Gender*				
Female	20	54.1	17	50.0
Male	17	45.9	17	50.0
				
*Ethnicity group*				
White British	28	75.7	28	82.4
Ethnic minority	9	24.3	6	17.6
				
*Marital status*				
Single	18	48.6	17	50.0
Married or cohabiting	17	45.9	14	41.2
Divorced	2	5.4	2	5.9
Widowed	0	0	1	2.9
				
*Employment status*				
Employed	26	70.3	24	70.6
Unemployed	0	0	1	2.9
Student	7	18.9	5	14.7
Retired	4	10.8	4	11.8
				
	n	%	n	%
*Traumatic event*				
DSM-IV PTSD criterion A1	37	100	34	100
Experienced event	37	100	34	100
Witnessed event	0	0	0	0
Brought in by ambulance	29	78.4	25	73.5
Type of motor vehicle accident				
Car/van/bus driver	19	51.4	13	38.2
Car/van passenger	0	0	4	11.8
Motorcyclist	6	16.2	5	14.7
Cyclist	12	32.4	8	23.5
Pedestrian	0	0	4	11.8
Perceived life threat to self (score>0)	31	83.8	31	91.2
Perceived life threat to other (score>0)	16	43.2	19	55.9
				
	*Mean*	*S.d.*	*Mean*	*S.d.*
Perceived life threat to self	5.19	3.20	5.56	3.23
Perceived life threat to someone else	2.22	3.25	3.56	3.99
Time since traumatic event (min)	192	69	211	67
Injury Severity Score	1.46	2.34	1.97	2.10
PDEQ score	19.86	8.02	19.18	8.40
PDI score	18.70	10.36	16.59	10.34
				
	n	%	n	%
*Treatment in emergency department*				
Location in emergency department				
Resuscitation	8	21.6	6	17.6
Majors	11	27.9	15	44.1
Minors/other	18	48.6	13	38.2
Admitted as in-patient	10	27.0	10	29.4
Received opiate medication	8	21.6	9	26.5
				
	n	%	n	%
*History of trauma and mental illness*				
Prior psychological trauma	28	77.8	24	70.6
Current/past mental illness	6	16.2	6	17.6
Family history of mental illness	10	27.8	7	20.6
Number of previous emergency department attendances in last year				
0	31	83.8	26	76.5
1–4	6	16.2	8	23.5

Abbreviations: DSM-IV, Diagnostic and Statistical Manual of Mental Disorders, 4th Edition; PDEQ, Peritraumatic Dissociative Experiences Questionnaire; PDI, Peritraumatic Distress Inventory; PTSD, post-traumatic stress disorder.

**Table 2:** Intention-to-treat results for primary and secondary outcomes in the trial

*Continuous outcome*	*Intervention (*n*=37)*		*Control (*n*=34)*		*Analysis*		
	*Mean*	*S.d.*	*Mean*	*S.d.*	t^a^	d	*95% CI for* d
*Primary outcome, 1 week*							
Number of intrusive memories of traumatic event	8.73	11.55	23.26	32.99	2.80**	0.67	0.18, 1.14

*Secondary outcomes, 1 week*							
Impact of Event Scale—Revised							
Intrusion subscale	7.27	5.27	10.70	7.29	2.25*	0.54	0.06, 1.01
Avoidance subscale	7.69	8.11	8.07	7.90	0.26	0.06	−0.41, 0.53
Hyperarousal subscale	5.26	5.79	6.98	7.42	0.96	0.23	−0.24, 0.70
Total	20.85	19.92	25.73	21.21	1.11	0.26	−0.21, 0.73
Post-traumatic Diagnostic Scale	11.38	8.55	14.28	11.94	0.83	0.20	−0.27, 0.67
Hospital Anxiety and Depression Scale	7.96	6.27	9.83	8.43	0.61	0.15	−0.32, 0.61

*Secondary outcomes, 1 month*							
Impact of Event Scale—Revised							
Intrusion subscale	5.21	5.09	7.01	6.90	0.93	0.22	−0.25, 0.69
Avoidance subscale	4.80	6.21	4.87	6.64	0.01	0.00	−0.47, 0.47
Hyperarousal subscale	4.29	6.47	5.28	6.45	0.59	0.14	−0.33, 0.61
Total	14.47	15.09	17.32	20.39	0.46	0.11	−0.36, 0.58
PDS	9.54	9.20	10.21	11.26	0.29	0.07	−0.40, 0.54
Hospital Anxiety and Depression Scale	7.44	7.20	8.12	8.20	0.18	0.04	−0.42, 0.51
							
*Categorical outcome, 1 month*	n	%	n	%	β^b^	*OR*	*95% CI for OR*
PDS symptoms consistent with PTSD criteria	4	12.9	3	9.7	0.34	1.4	0.28, 7.09

Abbreviations: CI, confidence interval; OR, odds ratio; PDS, Post-traumatic Diagnostic Scale; PTSD, post-traumatic stress disorder.

*P<0.05; **P<0.01.

a d.f.=69.

b Logistic regression, d.f.=1.
