# Supplementary material for: Preventing intrusive memories after trauma via a brief intervention involving Tetris computer game play in the emergency department: a proof-of-concept randomized controlled trial
Source: Mol Psychiatry. 2017 Mar 28;23(3):674–82. doi: 10.1038/mp.2017.23 (PMC5822451; doi:10.1038/mp.2017.23)
Supplement: Supplementary Information [file mp201723x1.docx]

**Supplementary Information**

Iyadurai, L., Blackwell, S. E., Meiser-Stedman, R., Watson, P. C., Bonsall, M. B., Geddes, J. R., Nobre, A. C. & Holmes, E. A. (2017). Preventing intrusive memories after trauma via a brief intervention involving Tetris computer game play in the emergency department: a proof-of-concept randomized controlled trial. *Molecular Psychiatry.* doi. 10.1038/mp.2017.23

**Reasons patients did not meet eligibility criteria**

**Supplementary Table 1.** Reasons Patients Did Not Meet Eligibility Criteria of Those Screened (n=505) and Those Assessed for Eligibility (n=2) Combined (Total n=507)

| Reason patient did not meet screening criteria | n |
| --- | --- |
| Could not be seen within 6 hours of leaving scene of accident | 166 |
| Insufficient physical mobility to play computer-game | 108 |
| Under 18 | 53 |
| Did not meet DSM-IV criterion A1 | 39 |
| No memory of accident | 24 |
| Glasgow Coma Scale (GCS) score < 15 | 21 |
| Not fluent in English | 15 |
| Neurological condition | 12 |
| Loss of consciousness > 5 mins | 6 |
| Intoxicated | 4 |
| History of severe mental illness | 3 |
| Suicidal | 2 |
| Substance abuse | 1 |
| Not able to complete study procedures (no glasses; deaf; migraine; drowsy from medication; insufficient time to be seen before discharge) | 53 |
| TOTAL | 507 |

Abbreviations: DSM-IV = Diagnostic and Statistical Manual of Mental Disorders, 4^th^ Edition.

**Supplementary Materials and Methods**

**Trauma memory “hotspots”**

“Hotspots” relate to the content of the intrusive memories – that is, particular parts within a traumatic experience that are described as the worst moments and are the contents of the intrusive sensory memories ([1](#_ENREF_1)). They were noted in the daily diary. Supplementary Table 2 shows a similar number of different trauma hotspot moments in the intervention and control groups, with examples of content.

**Supplementary Table 2.** Details of trauma memory hotspots noted in the daily diary in the intervention and control conditions (n=71)

|  | Intervention  (n = 37) | Control  (n = 34) |
| --- | --- | --- |
|  |  |  |
| Number of different hotspot moments within trauma, *M* (*SD*)  Range | 2.38 (1.01)  1 - 6 | 2.32 (1.04)  1 – 5 |
| Examples | Flash of airbag  Hearing sirens  Seeing smoke – being trapped  Hitting the bus  Losing control of the bike  Blood streaming/dripping  Catapulting forward  Dust and smoke, debris everywhere  Smell burning rubber  Windscreen smashing, darkness | Bang of airbags – white  Hearing bang – noise of crash  Seeing van indicator lights  Truck coming towards me – impact  Losing control of steering  Blood dripping on yellow raincoat  Car spinning  Curled up on all fours  Being dragged  Sparkly rain (glass) |

**Additional details regarding treatment conditions**

Intervention: The mean total duration of Tetris game-play in the Emergency Department (excluding a brief practice) was 18.5 minutes (SD = 4.2), and 7 participants (19%) took a brief break during gameplay.

Control: The mean duration of the activity log completion period was 22.4 minutes (SD = 15.1). The mean number of activities recorded in the log was 8.3 (range 3 to 21). Examples of recorded activities were “spoke to family”, “scan of chest + abdomen”, “bought coffee”, “was talking with my friend”, “nurse sorted out drip” and “drinking tea + eating biscuits”.

The total duration of Tetris game-play (intervention condition) and duration of the activity log completion period (control condition) did not differ significantly: Mann-Whitney U = 566.00, p = .46.

Total time spent in the Emergency Department did not differ significantly between the two conditions: intervention condition M = 3hrs 9mins, SD = 1hr 18mins, n = 35; control condition M = 3hrs 5mins, SD = 59mins, n = 32; t(65) = 0.142, p = .888^[[1]](#footnote-1)^.

Study materials in both conditions used the title “SCARTA - Simple cognitive activities after a road traffic accident”. All participants were told in the “Information sheet” that the study objective was “to examine how simple activities affect flashbacks and other symptoms after a road traffic accident”. The word “Tetris” was not used in the Information sheet, and the activity log used for the control condition was labelled “Activity diary”.

**Supplementary Results**

**Diary completion accuracy ratings**

Diary completion accuracy ratings (0-10) were high did not differ significantly between conditions: intervention 8.0 (SD = 1.9), control 8.5 (SD = 1.3), Mann-Whitney U = 476.00, p = .27.

**Attrition**

There were no differences in attrition between the intervention and control conditions at either time point (two-tailed Fisher’s exact test at one week p = .615, 8% vs 3%; at one month p = .482, 16% vs 9%, respectively).

**Per protocol analysis of primary and secondary outcomes**

The per protocol sample was pre-defined in the statistical analysis plan (https://osf.io/e4hc7) as all participants who received an adequate “dose” of the cognitive task intervention (if they were in the intervention condition), i.e. a memory reminder cue followed by playing Tetris for at least one uninterrupted period of 10 minutes, and who completed the outcome measures for one week follow-up (“follow-up 1”) and one month follow-up (“follow-up 2”) in the following time-frames: less than one month for follow-up 1, to assess acute stress symptoms, and from one month for follow-up 2, to assess post-traumatic stress symptoms. Results of the per protocol analyses are shown in Supplementary Table 3.

**Supplementary Table 3.** Per protocol results for primary and secondary outcomes

|  | Intervention  (n = 34) | | Control  (n = 33) | | Analysis | | |
| --- | --- | --- | --- | --- | --- | --- | --- |
| Continuous Outcome | Mean | SD | Mean | SD | t^1^ | d | 95% CI for d |
| Primary outcome, one week |  |  |  |  |  |  |  |
| Number of intrusive memories of  traumatic event | 8.68 | 11.22 | 23.52 | 33.73 | 2.73** | 0.67 | 0.17, 1.16 |
| Secondary outcomes, one week |  |  |  |  |  |  |  |
| Impact of Event Scale - Revised |  |  |  |  |  |  |  |
| Intrusion subscale | 7.26 | 5.29 | 10.73 | 7.42 | 2.21* | 0.54 | 0.05, 1.02 |
| Avoidance subscale | 7.50 | 7.16 | 8.06 | 8.07 | 0.15 | 0.04 | -0.44, 0.52 |
| Hyperarousal subscale | 5.06 | 5.54 | 7.00 | 7.60 | 1.03 | 0.25 | -0.23, 0.73 |
| Total | 19.82 | 16.66 | 25.79 | 21.4 | 1.23 | 0.30 | -0.18, 0.78 |
| Posttraumatic Diagnostic Scale | 11.24 | 8.26 | 14.27 | 11.9 | 1.21 | 0.30 | -0.19, 0.78 |
| Hospital Anxiety and Depression Scale | 8.03 | 5.92 | 9.88 | 8.56 | 0.53 | 0.13 | -0.35, 0.61 |
| Secondary outcomes, one month | N = 31 | | N = 30 | | t^2^ | d | 95% CI for d |
| Impact of Event Scale - Revised |  |  |  |  |  |  |  |
| Intrusion subscale | 5.26 | 4.77 | 6.23 | 5.73 | 0.52 | 0.13 | -0.37, 0.63 |
| Avoidance subscale | 4.90 | 6.05 | 4.17 | 5.11 | 0.47 | 0.12 | -0.38, 0.62 |
| Hyperarousal subscale | 4.06 | 4.70 | 4.43 | 5.21 | 0.25 | 0.06 | -0.44, 0.56 |
| Total | 14.23 | 14.09 | 14.83 | 15.02 | 0.13 | 0.03 | -0.47, 0.53 |
| Posttraumatic Diagnostic Scale | 9.61 | 8.82 | 8.83 | 8.28 | 0.01 | 0.00 | -0.50, 0.50 |
| Hospital Anxiety and Depression Scale | 7.52 | 6.51 | 7.43 | 7.31 | 0.23 | 0.06 | -0.44, 0.56 |
| Categorical Outcome | N | % | N | % | β ^3^ | OR | 95% CI for OR |
| PDS symptoms consistent with PTSD criteria | 4 | 12.9 | 3 | 10.0 | -0.29 | 0.75 | 0.15, 3.68 |

Abbreviations: PDS = Posttraumatic Diagnostic Scale; PTSD = posttraumatic stress disorder. Continuous outcomes were transformed to correct for skewness using the natural logarithmic function with the exception of the 1-week Impact of Event Scale—Revised intrusion subscale score (which approximated a normal distribution and thus no transformation was used) and the 1-week Posttraumatic Diagnostic Scale (for which a square root transformation was used).

^1^ df = 65; ^2^ df = 59; ^3^ Logistic regression, df = 1.

* p < 0.05, ** p < 0.01.

**Supplementary Discussion points**

**Supplementary Table 4.** Comparison of Study Sample (n = 71) and the Wider Population of Motor Vehicle Accident Admissions to the Emergency Department (ED) over the Study Period (n = 2298)

|  | Study sample  (n = 71) | | Wider population  (n = 2298) | | Comparison (between-groups) test | | |
| --- | --- | --- | --- | --- | --- | --- | --- |
|  | Mean | SD | Mean | SD | t | df | p |
| Age | 39.66 | 16.32 | 38.17 | 17.04 | 0.73 | 2367 | .47 |
|  | n | % | n | % | χ^2^ | df | p |
| Gender, female | 37 | 52.1 | 903 | 39.3 | 4.72 | 1 | .03 |
| Brought in by ambulance | 54 | 76.1 | 1175 | 51.1 | 17.13 | 1 | < .001 |
| Location in ED  Resuscitation  Majors  Minors/Other | 14  26  31 | 19.7  36.6  43.7 | 299  475  1524 | 12.6  20.1  67.3 | 17.69 | 2 | < .001 |

**Notes on feasibility of implementing the intervention in a hospital Emergency Department**

The feasibility of implementing the intervention ([2](#_ENREF_2)) is promising given the following: it can be integrated into wait times *around* standard medical care; delivered flexibly in many ED locations from bed to waiting area; does not require specialist training or a mental health professional for delivery but can be delivered by existing staff using a simple, manualised protocol; is simple and very brief (c. 20 minutes); does not require ongoing supervision; is low-cost - the only material resource is a game-play device, which could be the patient’s own smartphone; has no known adverse effects – critically, the intervention does not require the patient to talk about the trauma in detail which can be distressing ([c.f. 3](#_ENREF_3)); the computer game approach has broad appeal and may be seen as less stigmatising than seeing a mental health professional. In the current study, the intervention was met with enthusiasm and positive feedback from ED staff, who regarded this approach as feasible and practical.

**Expectancy ratings and primary outcome measure**

There was no significant relationship between participant expectancy ratings and their actual intrusive memory count either overall (n = 62, r = 0.11, p = .39; M = -0.07, SD = 4.07) or within each condition separately; intervention (n = 31*,* r = -0.16, p = .41; M = -1.48, SD = 4.66), control (n = 31, r = .27, p = .14; M = 1.35, SD = 2.79), with Fisher’s test showing no significant statistical difference in these relationships between groups (p > .10).

**References**

1. Holmes EA, Grey N, Young KAD. Intrusive images and "hotspots" of trauma memories in posttraumatic stress disorder: An exploratory investigation of emotions and cognitive themes. Journal of Behavior Therapy and Experimental Psychiatry. 2005;36(1):3-17.

2. Bird VJ, Le Boutillier C, Leamy M, Williams J, Bradstreet S, Slade M. Evaluating the feasibility of complex interventions in mental health services: standardised measure and reporting guidelines. The British Journal of Psychiatry. 2014;204(4):316-21.

3. Rothbaum BO, Kearns MC, Price M, Malcoun E, Davis M, Ressler KJ, et al. Early intervention may prevent the development of posttraumatic stress disorder: a randomized pilot civilian study with modified prolonged exposure. Biological Psychiatry. 2012;72(11):957-63.

1. Data were missing from medical records for 2 participants in the intervention condition and 2 participants in the control condition. [↑](#footnote-ref-1)
